# Psychological distress and digital health service use during COVID-19: A national Australian cross-sectional survey

**DOI:** 10.3389/fpsyt.2022.1028384

**Published:** 2022-10-20

**Authors:** Louise A. Ellis, Genevieve Dammery, Leanne Wells, James Ansell, Carolynn L. Smith, Yvonne Tran, Jeffrey Braithwaite, Yvonne Zurynski

**Affiliations:** ^1^Centre for Healthcare Resilience and Implementation Science, Australian Institute of Health Innovation, Macquarie University, Sydney, NSW, Australia; ^2^NHMRC Partnership Centre in Health System Sustainability, Australian Institute of Health Innovation, Macquarie University, Sydney, NSW, Australia; ^3^Consumers Health Forum of Australia, Canberra, ACT, Australia

**Keywords:** COVID-19 pandemic, mental health, psychological distress, digital health, telehealth

## Abstract

**Background:**

Previous research suggests that the COVID-19 pandemic caused significant disruption to the lives and mental health of Australians. In response, health services adapted rapidly to digital modes of treatment, prevention and care. Although a large amount of research emerged in the first year of the pandemic, the longer-term mental health impacts, contributing factors, and population-level utilization of digital health services are unknown.

**Methods:**

A population-based online survey of 5,100 Australians adults was conducted in October 2021. Psychological distress was assessed with the Kessler 6-item Psychological Distress Scale. Additional survey questions included use and satisfaction with digital health services. Where available, data were compared with our previous survey conducted in 2018, permitting an examination of pre- and post-pandemic digital health service utilization.

**Results:**

In 2021, almost a quarter (*n* = 1203, 23.6%) of respondents reported serious levels of psychological distress; participants with pre-existing health related conditions, of younger age, lower educational attainment, those who lost their job or were paid fewer hours, or living in states with lockdown policies in place were at highest risk of serious psychological distress. Almost half of all respondents (*n* = 2177, 42.7%) reported using digital health technologies in 2021, in contrast to just 10.0% in 2018. In 2021, respondents with serious psychological distress were significantly more likely to consult with a healthcare professional via telephone/videoconferencing (*P* < 0.001), access healthcare via a telephone advice line (*P* < 0.001), or via an email or webchat advice service (*P* < 0.001) than those with no serious psychological distress. Those with and without psychological distress were highly satisfied with the care they received via digital health technologies in 2021.

**Conclusion:**

Rates of serious psychological distress during the second year of the pandemic remained high, providing further evidence for the serious impact of COVID-19 on the mental health of the general population. Those with psychological distress accessed digital mental health services and were satisfied with the care they received. The results highlight the continued need for mental health support and digital health services, particularly for people living with chronic conditions, younger adults and people most impacted by the COVID-19 pandemic, both in the short term and beyond.

## Introduction

The adverse mental health consequences of COVID-19 were recognized early in the pandemic, with the World Health Organisation (WHO) declaring that addressing mental health needs must be an “integral part of the COVID-19 response”(1) (p.129). The situation was characterized as a “black swan moment”– an unforeseen event necessitating a robust shift in mental health care to the provision of digital mental health prevention, treatment and care (2). Recognizing the imperative to safeguard access to mental health services and respond to potentially increased demand, mental health services across the globe quickly shifted to new modes of delivery, in particular the use of digital mental health care (3).

### Mental distress and its contributing factors during COVID-19

Worldwide population health data has provided evidence to suggest that mental health significantly deteriorated across the globe in 2020 (3). In Australia, one study identified that the population prevalence of mental distress more than doubled from 20% (4) to around 45% in the first few months of the pandemic (5), with similar increases being reported in the United States and other countries (6, 7). Historically, research has also identified the mental health challenges that have accompanied previous pandemics (e.g., SARS outbreak of 2003), including reports of increased acute stress, anxiety and depression (8). Moreover, the widespread global lockdown restrictions adopted in response to COVID-19 are also thought to have exacerbated the likelihood of mental health challenges within the community by increasing social isolation and loneliness and decreasing family and social support (9).

Although there is widespread evidence for increased mental distress in the early months of the pandemic, much of the existing research has failed to account for the “heterogeneity in psychological response to the outbreak” (10), with a single profile of mental ill-health for the entire population being described as “unrealistic” (p.2). In support of a heterogeneous response, worldwide mental health data from the first year of the pandemic suggests that mental distress varied considerably across population groups; with the groups at highest risk including young people, those living alone, those with lower-socioeconomic status, and those who became unemployed during the pandemic (3). Two published studies from Australia during the first few months of the pandemic similarly identified being young, female, having financial stress, and having a prior mental health diagnosis as risk factors for poor mental health during the pandemic (11, 12). Hence, in our examination of mental distress in the Australian population, during the last quarter of 2021, we also sought to examine the contributing factors to poor mental health, in order to identify at-risk groups most in need of support.

### Disruption in mental health services during COVID-19

The introduction of lockdown restrictions also caused significant disruption in mental health services, with face-to-face care being reduced in favor of digital health services. A survey conducted by the World Health Organization (WHO) in the second quarter of 2020 found that more than 60% of countries worldwide reported disruptions to mental health services (13). By mid-2020, more than 80% of high-income countries had shifted to digital health technologies to replace or supplement face-to-face mental health consultations (3). In this paper, we define digital health (also often termed e-health) as services delivered online or via telephone (14). A subset of digital health, most commonly termed telehealth or telemedicine, historically focused on service provision via telephone, yet technological advancement has enabled telehealth to now be delivered via communication software including videoconferencing (15).

With the onset of COVID-19, many governments around the world have been responsive in ensuring the availability of digital health to their population, in some cases by adding new reimbursements for services (15). In Australia, in response to COVID-19, the government provided additional funded telehealth services through the Medicare Benefits Schedule, enabling a greater range of telehealth services to be re-imbursed, including telephone and videoconferencing with general practitioners and specialists (16). As a result, the proportion of healthcare consultations provided by videoconference is reported to have increased from 0.2% in February 2020 (prior to funding changes) to 35% provided by telephone and videoconference in April 2020 (17). Two published studies also pointed to the increase in demand for two specific telephone and online support services, Kids Helpline (Australia’s national youth helpline) and MindSpot (an Australian digital mental health clinic), in the early stages of the pandemic (18, 19). These studies identified users’ primary reasons for making contact, with these including concerns with the virus itself, mental health concerns, the effects of social isolation and financial insecurity. However, broader digital service use and satisfaction with received care at a population level remains unknown.

### The present study

The overarching objective of this study was to examine mental distress and digital health service use among Australian adults during the second year of the COVID-19 pandemic. A population-based online survey was conducted in October 2021, at a time when two of Australia’s most populous states (Victoria and New South Wales) had strict lockdown policies in place (see Box 1 for further information on Australia’s COVID-19 situation and response to the pandemic at the time). Where available, data were compared with previous population-based data collected in 2018, allowing for an examination of change in pre- and post-pandemic digital health service use. The specific aims of this study were to:

BOX 1 **Australia: COVID-19 situation and policy response.**

As at 2nd October 2021, a total of 109,315 cases of COVID-19 had been reported in Australia, including 1,321 deaths. The majority of cases were from New South Wales and Victoria.

A snapshot of case numbers and statistics can be found at: https://www.health.gov.au/sites/default/files/documents/2021/10/coronavirus-covid-19-at-a-glance-2-october-2021.pdf.

Australia’s national strategy of “aggressive suppression” involved the early application of strict lockdown restrictions when community transmission was observed, thus avoiding the spread of large waves of infections and deaths seen in many other parts of the world (20, 21).

Australia’s federal system meant that restriction decisions were made independently by the government of each state or territory, which resulted in some states enduring much harsher and sustained lockdowns than the remainder of Australia. During the time of this survey, New South Wales and Victoria had strict lockdown policies in place, including business closures, stay-at-home orders, remote schooling, and evening curfews in order to suppress community transmission. The other Australian states and territories were comparatively free of COVID-19 restrictions at the time.

(1) Investigate psychological distress and its contributing factors among the general Australian population in the second year of the pandemic (i.e., 2021).

(2) Compare the extent of digital health service use and user satisfaction during the second year of the pandemic (i.e., 2021), compared with pre-COVID-19 population data from 2018.

(3) To examine differences in digital health service use and satisfaction with care received through these modalities between psychologically distressed and non-psychologically distressed groups in 2021.

## Materials and methods

### Participants

Australian participants aged ≥18 years were recruited through a global market research company, Dynata, a leading international digital data collection company, with over 200,000 panelists registered from Australia. Dynata was contracted to recruit a sample of 5,000 Australians, based on representative quotas for age, gender and geographical location. Dynata’s sampling algorithm ensures random selection and demographic representation. To ensure reliability and accuracy of data, panel members undergo a rigorous verification process and incoming data undergoes various quality checks, including participation limits, digital fingerprinting, and removing panel members that provide illogical responses or do not spend sufficient time answering survey questions.

Participants were contacted in late September to early October 2021 via email and invited to participate in the online survey. The survey was open for a two-week period. Informed consent was provided by participants as part of the opt in process. Dynata uses a point system for survey participation that allows panel members to exchange their points for cash, airline miles, or other prizes. Ethics approval was granted by The Macquarie University Human Research Ethics Committee (Ref no: 5201836705403).

### Survey

The online survey was co-designed with researchers from the Australian Institute of Health Innovation and consumer researchers from the Consumers Health Forum of Australia, with additional feedback provided by Australian Government Department of Health. The survey included a total of 67 questions, which took an average of 20 minutes to complete. Several survey questions were consistent with those asked in the 2018 Australian Consumer Sentiment Survey (22, 23), providing a base for comparison. The focus of this paper is on items related to mental health status, digital health service use and satisfaction with care received through digital health modalities. We also collected data on a variety of demographic factors, including gender, age, postcode, and level of education, as well as the presence of chronic conditions, and COVID-19 related employment changes, such as job disruption and loss of income. The results of the remaining items will be published elsewhere. The survey was conducted in English only due to resource constraints.

#### Mental health status

The Kessler 6-item Psychological Distress Scale (K6) was developed as a brief screening tool to identify serious mental illness, and is now a well-established measure of psychological distress (24). It is most strongly associated with affective disorders (major depressive disorder, bipolar disorders, dysthymia) and anxiety (25). The K6 consists of six items that ask respondents about how they have felt over the past four weeks: nervous, hopeless, restless or fidgety, so depressed that nothing could cheer them up, that everything was an effort, and worthless. Response options range from “none of the time” (1) to “all of the time” (5). Scores are summed, with total scores ranging between 6 and 30 (Australian scoring) (26). We used the established cut-point of a score of 19 or higher to define a binary indicator of high levels of psychological distress and likely serious mental illness (24, 26).

#### Chronic conditions

Respondents were asked to select whether they had any of the following long-term chronic health conditions (defined as lasting six months or more): arthritis; asthma; back pain or back problems; cancers; cardiovascular disease; chronic obstructive pulmonary disease; diabetes; kidney disease; mental disorders; and/or osteoporosis; based on the Australian Institute of Health and Welfare’s (AIHW) major groupings and definitions of chronic conditions (27). Participants were asked to “select as many as applicable”, or to specify another condition, and those without a chronic condition chose: “No, none of the above.”

#### COVID-19 related employment changes

Respondents were asked whether they lost their job (no, yes) or worked fewer paid hours (no, yes) at any time during the COVID-19 pandemic. We restricted our analysis to these two key variables (i.e., lost job, less paid work), based on previous evidence showing the impact of lost work on mental health during COVID-19 in Australia (28).

#### Digital health service use

To assess the nature of digital health service use, participants were asked to indicate whether they have accessed healthcare over the previous 12 months via: telephone or video; telephone advice line (e.g., Lifeline, Beyond Blue); email or webchat helpline or adviceline (e.g., headspace online; no, yes). This question was also included in the previous 2018 Australian Consumer Sentiment Survey (22, 23).

#### Satisfaction with digital health

Those participants who indicated use of a digital health service were then asked how satisfied they have been with the care they received. Degree of satisfaction was rated on a 5-point Likert scale from “very dissatisfied” (1) to “very satisfied” (5). This question was included in the previous 2018 Australian Consumer Sentiment Survey (22, 23).

### Data analysis

Survey data collected in 2021 and 2018 were post-weighted by age, sex and state to reflect population distribution according to the Australian Bureau of Statistics (ABS) demographic statistics of March (29) and June (30), respectively. The two surveys were post-weighted through a survey raking technique using the anesrake package in R (31).

Postcode data was mapped to the Australian Statistical Geography Standard (ASGS) which allocates classes of remoteness to localities, based on the Accessibility/Remoteness Index of Australia (ARIA +): major cities, inner regional, outer regional, remote and very remote. For analysis, the five categories were recoded as a dichotomous variable: major city and regional/remote. Level of education was also recoded as a dichotomous variable: school (primary/secondary) and tertiary (technical college/university degree). Respondents who reported a chronic mental disorder were classified into a dichotomous variable (no, yes), and those who reported one or more of the other nine chronic health conditions were classified as having an ‘other chronic condition’ (no, yes). At the time of the survey, the states of Victoria (VIC) and New South Wales (NSW) were under severe lockdown restrictions, enabling us to examine the effect of the lockdown on mental health, with the Australian states being classified into a dichotomous variable (lockdown status): NSW/VIC and ‘all other states’. Finally, satisfaction with digital health services was recoded into two levels: low satisfaction (very dissatisfied, somewhat dissatisfied, neither satisfied nor dissatisfied) and high satisfaction (somewhat satisfied, very satisfied). All data transformations and analyses were conducted using IBM SPSS Statistics Version 27.0 (32).

The statistical analysis for each of the study aims is reported below. In addressing Aim 1, chi-square (χ^2^) analysis was used to examine differences in proportions of serious psychological distress for potential categorical predictor variables. Logistic regression models were then used to predict the odds of serious psychological distress, by incorporating independent variables that were statistically significant from univariate analyses. In Step 1, demographic independent variables included age (<45 years, 45 + years), gender (male, female), rurality (major city, rural/remote), and level of education (school, tertiary); Step 2 included pre-existing health related conditions: chronic mental disorder (no, yes) and other chronic condition/s (no, yes); and Step 3 included COVID-19 related employment status: lost job (no, yes), less paid work (no, yes); and lockdown status (other states, NSW/VIC). The results from the model have been expressed as odds ratios (95% confidence interval) and p-value for statistical significance.

To address Aim 2, chi-square analysis was used to examine differences in digital health service use and satisfaction across the two survey cohorts (2021 vs. 2018). Comparisons across the two surveys were only made where questions were identical. For Aim 3, chi-square analysis was used to examine differences in digital health service use and satisfaction between psychologically distressed and non-psychologically distressed groups. For all analyses, a *p*-value of <0.05 was considered to be statistically significant.

## Results

### Characteristics

In total, 5,100 Australians participated in the 2021 Australian Consumer Sentiment survey. Dynata did not provide the research team with the total number of contacts made to result in the final 5,100 respondents. Participants were aged between 18 and 99 years (M = 46.8, SD = 17.6), with 51% of the sample being female. Table 1 presents sample demographics (unweighted n’s and weighted percentages), along with a comparison of participant demographics from the previous 2018 Australian Consumer Sentiment survey. As shown, our post-weights were broadly successful in creating two datasets appropriate for comparison, taking into account differences in key demographics.

**TABLE 1:** Study participant characteristics across 2021 and 2018 surveys.

Characteristics		2021 *n*^a^ (%)^b^	2018 *n*^a^ (%)^b^
Overall		5,100	1,024

Gender	Male	2475 (49.0%)	432 (49.0%)
	Female	2576 (51.0%)	592 (51.0%)
Age	18-24 years	614 (12.0%)	68 (12.0%)
	25-44 years	1853 (36.3%)	352 (37.0%)
	45-64 years	1589 (31.2%)	383 (32.0%)
	65 years +	1043 (20.5%)	221 (19.0%)
State	ACT	86 (1.7%)	9 (2.0%)
	NSW	1623 (31.8%)	330 (32.0%)
	NT	49 (1.0%)	2 (1.0%)
	Qld	1033 (20.3%)	218 (20.0%)
	SA	351 (6.9%)	83 (7.0%)
	Tas	108 (2.1%)	22 (2.0%)
	Vic	1319 (25.9%)	262 (25.0%)
	WA	531 (10.4%)	98 (11.0%)

Numbers may not equal total sample due to missing values; ^a^Unweighted; ^b^Weighted for age, sex, and state.

### Aim 1: Investigate psychological distress and its contributing factors (2021 data)

The prevalence of serious psychological distress for the 2021 sample was 23.6% (*n* = 1,203). Table 2 shows the proportions of respondents reporting serious levels of psychological distress for key demographic, health and COVID-19 related variables of interest. Analysis by age groups indicated a significant and decreasing trend in serious psychological distress by age, χ^2^ (3) = 512.09, *p* < 0.001; with respondents in the 18 to 24 years (41.8%) and 25 to 44 years (34.9%) age groups reporting the highest levels of serious psychological distress. Analysis for gender indicated significantly higher prevalence of serious psychological distress for females (25.5%) than males (21.1%), χ^2^ (1) = 14.04, *p* < 0.001. Those with no formal educational qualifications beyond high school (38.1%) also had significantly higher prevalence rates for serious psychological distress than those with tertiary level qualifications (18.5%), χ^2^ (1) = 203.64, *p* < 0.001. Respondents with a pre-existing chronic mental disorder were also more likely to report serious psychological distress (45.8%), as were those with any other chronic condition/s (30.9%), than those without a chronic mental disorder (19.1%) or other chronic condition (15.2%). In terms of COVID-19 employment related factors, those who had lost their job (49.2%) or were paid less hours (36.3%), had significantly higher prevalence of serious mental distress than those who had not lost their job (20.6%) and were not being paid less hours (20.1%). Respondents living in VIC or NSW also had significantly higher levels of serious mental distress (26.0%) than those living in other states (20.3%), χ^2^ (1) = 23.13, *P* < 0.001. There was no significant difference in the prevalence of mental distress for those living in major cities (22.9%) compared to those living in rural/remote areas (24.6), χ^2^ (1) = 1.96, *P* = 0.16.

**TABLE 2:** Prevalence of serious psychological distress across demographics, health and pandemic-specific factors.

Variable		Serious psychological distress, *n (%)*	Chi-square, χ^2^ (df)	*P-value*
Gender	Male	522 (21.1)	14.04 (1)	<0.001**
	Female	658 (25.5)		
Age	18-24 years	257 (41.8)	512.09 (3)	<0.001**
	25-44 years	647 (34.9)		
	45-64 years	257 (16.2)		
	65 years +	43 (4.1)		
Rurality	Capital city	682 (22.9)	1.96 (1)	0.16
	Regional/Remote	521 (24.6)		
Education	School	488 (38.1)	203.64 (1)	<0.001**
	Tertiary	698 (18.5)		
Chronic mental disorder	No	813 (19.1)	279.49 (1)	<0.001**
	Yes	390 (45.8)		
Other chronic condition	No	361 (15.2)	173.92 (1)	<0.001**
	Yes	842 (30.9)		
Lost job	No	944 (20.6)	213.50 (1)	<0.001**
	Yes	258 (49.2)		
Less paid hours	No	805 (20.1)	125.53 (1)	<0.001**
	Yes	398 (36.3)		
State	All other States	437 (20.3)	23.13 (1)	<0.001**
	NSW/VIC	766 (26.0)		

*P < 0.05, ** *P* < 0.001. Significant values have been bolded.

The results from the multivariate logistic regression for the prediction of serious psychological distress is displayed in Table 3. Since rurality was not found to be significantly different for serious psychological distress in the univariate chi-square result, this variable was not entered into the multivariate logistic regression model. In Step 1, the demographic predictor variable model, including age (45 + years, < 45 years), gender (male, female) and level of education (tertiary, school), was significant (χ^2^(3) = 555.52, *P* < 0.001), accounting for 15.9% of the variance in psychological distress. Step 2 included the addition of pre-existing health related conditions, chronic mental disorder (no, yes) and other chronic condition/s (no, yes), accounting for a significant additional 12.8% of the variance (**Δ**χ^2^(2) = 86.34, *P* < 0.001). Step 3 included COVID-19 related employment status, lost job (no, yes), less paid work (no, yes) and lockdown status (other States, VIC/NSW), accounting for a significant additional 2.1% of the variance (**Δ**χ^2^(3) = 86.34, *P* < 0.001).

**TABLE 3:** Predictors associated with serious psychological distress.

Variable		*b (SE)*	OR (95% CI)	*P-value*
**Step 1**				
Gender	Male ^a^			
	Female	0.065 (0.079)	1.067 (0.913-1.247)	0.41
Age	45 years + ^a^			
	18-44 years	1.428 (0.087)	4.171 (3.515-4.950)	<0.001**
Education	Tertiary ^a^			
	School	0.813 (0.087)	2.255 (1.903-2.672)	<0.001**
**Step 2**				
Chronic mental disorder	No ^a^			
	Yes	1.489 (0.094)	4.432 (3.686-5.329)	<0.001**
Other chronic condition	No ^a^			
	Yes	1.031 (0.083)	2.804 (2.385-3.296)	<0.001**
**Step 3**				
Lost job	No ^a^			
	Yes	0.819 (0.111)	2.269 (1.825-2.820)	<0.001**
Less paid hours	No ^a^			
	Yes	0.383 (0.088)	1.467 (1.236-1.742)	<0.001**
State	All other States ^a^			
	NSW/VIC	0.192 (0.080)	1.212 (1.037-1.417)	<0.05*

^a^Reference Category; OR = Odds Ratio; CI = Confidence interval; *P < 0.05, **P < 0.001. Significant values have been bolded.

The multivariate model suggested that age, education, chronic mental disorder, other chronic condition/s, lost job, working less paid hours and living in NSW or VIC, were all significant and unique factors in predicting serious psychological distress during the last quarter of 2021. Odds ratios demonstrate that younger respondents (18 to 44 years) were four times more likely to have serious mental distress than older respondents (45 + years) (OR = 4.17, 95% CI 3.52-4.95). Those with school education were twice as likely to report serious psychological distress than those with tertiary education (OR = 2.26, 95% CI 1.90-2.67). Respondents with a chronic mental disorder were more than four times as likely to report serious mental distress (OR = 4.43, 95% CI 3.69-5.33), and those with other chronic conditions were almost three times as likely to report serious mental distress than those without (OR = 2.80, 95% CI 2.39-3.30). In terms of COVID-19 related factors, participants who had lost their job or were being paid fewer for hours of work during the pandemic were two and 1.5 times more likely to report serious mental distress, respectively. Finally, respondents living in VIC or NSW had 21% higher odds of having reported serious mental distress compared to those living in other states (OR = 1.21, 95% CI 1.04-1.42). After adjusting for other predictors in the model at Step 2, gender was no longer significant.

### Aim 2: Comparison of 2021 digital health service use and user satisfaction rates with pre-COVID 2018 data

**FIGURE 1:**
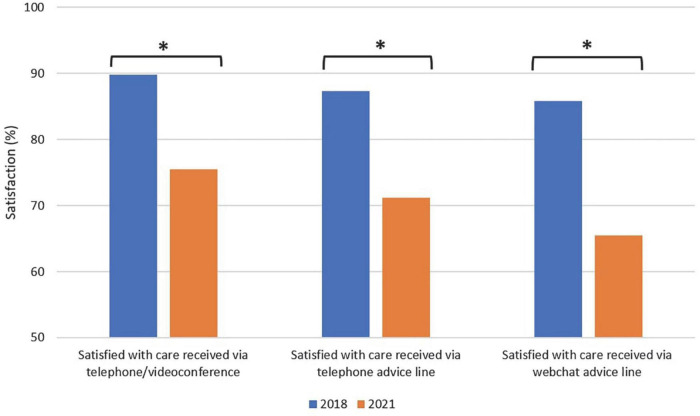
Satisfaction with digital health services in 2018 and 2021. *P < 0.05, **P < 0.001.

Overall, digital health use reported in 2021 was significantly higher than 2018 (42.7% vs. 10.0%, χ^2^ (1) = 391.27, *P* < 0.001) among the general population. Specifically, reported utilization in 2021 was significantly higher than 2018 for telephone/videoconferencing (37.1% vs. 5.2%, χ^2^ (1) = 401.34, *P* < 0.001), telephone advice line (11.6% vs. 5.1%, χ^2^ (1) = 38.82, *P* < 0.001) and email/webchat helpline (6.6% vs 2.8%, χ^2^ (1) = 21.98, *P* < 0.001). In 2021, a higher proportion of respondents reported high levels of satisfaction with the care they received via: telephone/videoconferencing compared with 2018 (89.8% vs. 75.5%, χ^2^ (1) = 11.18, *P* = 0.001), telephone advice line (87.4% vs. 71.2%, χ^2^ (1) = 10.55, *P* = 0.001) and webchat advice line (85.8% vs. 65.5%, χ^2^ (1) = 8.31, *P* = 0.004; also see Figure 1).

### Aim 3: Examine digital health service use and satisfaction with care by mental health status (2021 data)

**FIGURE 2:**
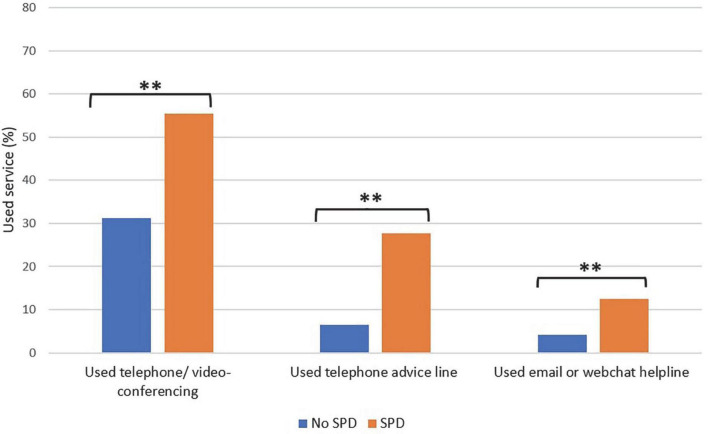
Digital health service use by mental health status in 2021. **P < 0.001, SPD = serious psychological distress.

Respondents with serious psychological distress in 2021 were significantly more likely to consult with a healthcare professional via telephone/videoconferencing (55.4% vs. 31.2%, χ^2^ (1) = 23.13, *P* < 0.001), access healthcare via a telephone advice line (27.7% vs. 6.4%, χ^2^ (1) = 414.64, *P* < 0.001), or via an email or webchat advice line (14.1% vs. 4.2%, χ^2^ (1) = 148.90, *P* < 0.001) than those with no serious psychological distress (also see Figure 2).

Overall, for the total sample of 5,100, respondents reported high levels of satisfaction with the care they received via telephone/videoconferencing (*n* = 1889, 89.8%), telephone advice line (*n* = 567, 87.4%) and webchat advice line (*n* = 324, 85.8%). Those with serious psychological distress reported higher levels of satisfaction with the care they received compared with those without serious psychological distress, though the differences were not significant: telephone/videoconferencing (91.5 vs. 88.8%, χ^2^ (1) = 3.28, *P* = 0.070); telephone advice line (88.4 vs. 86.1%, χ^2^ (1) = 0.69, *P* = 0.406); webchat advice line (86.4 vs. 85.3%, χ^2^ (1) = 0.08, *P*.774).

## Discussion

From this demographically representative survey conducted in the second year of the COVID-19 pandemic, we identified that almost one in four adults exhibited serious psychological distress (23.6%); a result that is concordant with comparable measures of mental distress in the Australian population in the first year of the pandemic. For example, the *Taking the Pulse of the Nation survey 2020*, reported rates of mental distress between 20 and 25% during 2020, over double the rate of mental distress in the Australian community prior to the pandemic (10%) (33). Similar increases in mental distress have also been reported in the United States and Europe (3, 6, 34). In research on large samples of adults in the United States, depression symptom prevalence more than doubled in the first months of the COVID-19 pandemic; 25.8% of the population showed symptoms of moderate depression or greater, compared to 8.5% before the pandemic (6). Reports of increases in psychological distress have not only been restricted to high-income countries, with mental health impacts also being identified in lower middle-income countries, such as Vietnam (35) and Egypt (36).

Our results are also broadly congruent with repeated cross-sectional data on measures of mental health and wellbeing throughout the pandemic. The Australian National University’s *COVID-19 Impact Monitoring Survey Program*, a quarterly probability-based panel survey of public opinion run throughout 2020 and 2021, showed peaks in psychological distress early in the pandemic (mostly aligned with Australia’s lockdowns), with elevated levels around April and October 2020 and again in October 2021. The latter peak has been reported to likely reflect that Australia’s two largest states had lockdown restrictions in place, and also corresponds with the time when this survey was conducted (37).

### High-risk groups for psychological distress

An identified limitation of previous studies investigating the mental health impact of the pandemic is their focus on overall “mean effects without addressing the possibility of heterogeneity in mental health” response (p.2) (38). However, in this study we sought to examine contributing factors to mental distress in order to identify sub-groups of individuals most at risk and in need of support. Our results identified heightened risk for the following groups: those with a pre-existing health related condition, those of younger age or lower educational attainment, those who lost their job or were paid fewer hours, or those living in states with strict lockdown policies in place. Although much of the existing research did not consider a broad range of predictors, two other Australian studies conducted during the first months of the pandemic also showed higher risk for younger adults, those with a pre-existing mental health condition, and those experiencing financial distress (11, 12). In line with previous research (11, 12), we also found heightened risk for mental distress for females; however, the level of risk did not persist when other factors such as age, gender and chronic condition were taken into account.

Although there is previous evidence to suggest that lockdowns have adverse psychological consequences, much of this research was conducted in the early stages of the pandemic and/or used proxy measures of mental health (e.g., calls to telephone hotlines), leaving the effects of sustained lockdowns unknown. Furthermore, Australia has been identified as a unique context for examining the impact of sustained lockdowns (39). Firstly, Australia pursued a national “aggressive suppression” strategy involving the early application of strict lockdown restrictions to avoid the consequences of the large waves of infections seen in many other parts of the world (20, 21). Secondly, Australia’s federal system meant that restriction decisions were made independently by the government of each state or territory, which resulted in some states enduring much harsher and sustained lockdowns (i.e., Victoria and New South Wales) than the remainder of Australia; this provides a natural comparison group for research. In fact, Victoria enacted six lockdowns, totaling 262 days (40), receiving the title of the “longest lockdown in the world” in October 2021 (41). Thus, this study presented a unique opportunity to examine the impact of lockdowns on mental health. Indeed, we identified that those living in states with lockdown policies in place had 21% higher odds of reporting serious mental distress. However, concordant with other international research during the pandemic, our study suggests that lockdowns are unlikely to have uniformly detrimental effects (42), with other factors such as age, education, health-related factors and financial situation contributing to the risk of psychological distress.

Notably, in this study, respondents with chronic conditions were more than three times as likely to report serious psychological distress than those without. The significant and substantial influence of pre-existing chronic conditions is unsurprising given widespread public awareness that people with chronic conditions (e.g., diabetes, chronic obstructive pulmonary disease, heart disease) are disproportionately prone to COVID-19 related hospitalizations, intensive care admissions and mortality, with warnings to this effect being incorporated in national public health guidelines (43). It is also likely that people with chronic conditions may have been particularly anxious due to difficulties in accessing necessary health services during the pandemic (44). For those with a chronic mental health condition in particular, the pandemic may have precipitated feelings of fear, anxiety and panic, thus exacerbating mental health symptoms. Being in lockdown, for example, jeopardizes daily routine and social rhythm, thereby increasing stress and escalating cortisol levels, and potentially resulting in an exacerbation of depressive and/or anxiety symptoms (45). Further, barriers in accessing mental health services increases the risk of mental health symptom exacerbation and relapse.

Young Australians are also reported to have been disproportionately impacted by the pandemic. Compared with older age groups, young people have experienced higher rates of loneliness, educational disruption, unemployment, housing distress and domestic violence (46). The closure of schools and universities has disrupted friendship and support networks for young people (47). Previous research has also identified that young adults feel most stressed by uncertainty, such as not knowing when the pandemic will end (48). While some of the effects of COVID-19 on young people are emerging, “the full impact on them is complex and not yet fully understood” (46), including the need to examine the longer-term outcomes for young people (e.g., consequences of remote working on longer term employment, career progression and social skills).

Our results align with a study undertaken in the early stages of the pandemic which found that groups experiencing job loss were up to three times as likely to experience high psychological distress (28). With the casualization of the Australian workforce prior to the pandemic, casual and part-time workers who are more likely to be young people or women, were more likely to lose work, or were left without entitlements such as access to paid sick leave. In addition, casual workers were 8 times more likely to lose work in the 2021 lockdowns than permanent staff, leaving such workers extremely vulnerable given that their weekly earnings are 52% lower than for permanent employees (49).

### Digital health service use

In this study, almost half of all respondents reported using digital health technologies in 2021, an increase from just 10.0% in 2018. The substantial increase in digital health use following the onset of the pandemic was expected given that digital health technologies were propelled into widespread use following the temporary closure of face-to-face mental health services (15). Nevertheless, significant increases in utilization were not only identified for telehealth services, but also for telephone advice lines and email or webchat services. Moreover, respondents with psychological distress were significantly more likely to access all forms of digital health technologies than those without psychological distress. Indeed, the *Household Impacts of COVID-19 Survey* conducted by the Australian Bureau of Statistics in May (29) identified almost three quarters (72%) of Australians reported using one or more strategies to manage their mental health since the start of the pandemic (50). Analysis of Australia’s Medicare Benefits Schedule data also shows that there was an uptrend in the use of telehealth services from later May 2021 through October 2021 corresponding with the sustained lockdowns in VIC and NSW (51). The vast majority of respondents (> 85%) also reported high levels of satisfaction with all forms of digital health technologies, which is supported by other studies reporting high levels of satisfaction specifically with telehealth internationally (52). Together, these findings support the use of digital health tools for delivering health care, with the general public utilizing digital health tools and being highly satisfied with the care they receive.

### Implications and future research

With such high numbers of the Australian population with elevated psychological distress, digital health services represent a powerful option to support the wellbeing of communities and offset the difficulties in meeting rising mental health demands through existing face-to-face services. The good news is that, across Australia, the potential mental health consequences of the pandemic were recognized early, with digital health service delivery widely embraced by clinicians and the general public. For 2022, the focus has been upon planning for the post-COVID world and returning to normalcy. Yet, since the most recent outbreaks across Australia “that feeling has turned to dismay”, “with the nation tilting into anxiety, unsure of its economy, and tightly bound to world events outside its control” (53). We are not out of the woods yet.

Our study highlights the value of conducting surveys of mental health status and needs periodically, particularly as we emerge from the COVID-19 pandemic. Longitudinal studies of the high-risk groups of mental distress identified in this study would be particularly beneficial, monitoring their trajectories and use of digital health services. As we emerge from COVID-19, the key question is whether digital services are likely to remain the “new normal”. Given that many of the digital health tools are adopted by consumers outside of the usual health care system, how can we optimize their integration into existing health care models and systems?

### Strengths and limitations

A unique strength was the repeated cross-sectional data for a number of questions at two time points: pre- and post-pandemic. The samples were demographically representative for age groups, gender and geographical distribution across the two time points, and the sample sizes were large enough to support statistical confidence and power. Health consumer representatives from the Consumers Health Forum of Australia participated in the co-design and deployment of this survey, and provided vital advice about analysis and interpretation of results. However, there were limitations to the comparisons over time, as not all questions were asked at the two time points, most notably mental health status. We also acknowledge that this study was cross-sectional and responses were self-reported, thus, the direction of associations cannot be determined and results may be affected by response and sampling bias. To avoid issues with survey fatigue, the number of survey questions was kept to a minimum and did not include questions about living situation (e.g., live alone or with others). As there is evidence to suggest that household situation can impact mental health status, we will aim to include this question in our next national survey. We will also include more in-depth questions about digital health service use (e.g., frequency and purpose of use) in the next survey round. Further, due to limited resources, the survey was conducted in English, thus potentially preventing non-English speakers from completing the survey.

We were unable to establish a survey response rate because of the sampling process applied to an established panel and we have no details on non-respondents. Further, although the purposeful sample was matched to the Australian Population in terms of age, gender and geographical distribution, consumer panels can be subject to bias and may not be truly representative of the general population. In particular, given that the surveys are completed online, panel members are likely to have higher digital literacy and socioeconomic status compared to the general population, particularly amongst older adults (54). Our participants also share the common characteristic of being willing to participate in survey research, which may bias the results.

## Conclusion

The pandemic has provided an unprecedented impetus to propel digital health services into widespread use, and this study explored their utilization and satisfaction at this crucial point in time. Our understanding of the magnitude of psychological distress in relation to the COVID-19 pandemic is growing. Healthcare systems in Australia and around the world have sought to meet the rising mental health needs by facilitating access and encouraging the use of digital health services. In this study, we observed a robust increase in utilization of digital health services; those with psychological distress were even more likely to access digital health services and were satisfied with their use. The results highlight the continued need for mental health support and access to digital health services, particularly for younger adults and those most impacted by the COVID-19 pandemic, both in the short term and beyond.

## Data availability statement

The raw data supporting the conclusions of this article will be made available by the authors, without undue reservation.

## Ethics statement

The studies involving human participants were reviewed and approved by Macquarie University Human Research Ethics Committee (Ref no: 5201836705403). The patients/participants provided their written informed consent to participate in this study.

## Author contributions

LE, GD, CS, JB, LW, JA, and YZ conceptualized the study. LE analyzed the data with input from YT. LE drafted the manuscript with input from GD. All authors read and approved the final manuscript.
